# Novel Insight Into the Etiology of Autism Spectrum Disorder Gained by Integrating Expression Data With Genome-wide Association Statistics

**DOI:** 10.1016/j.biopsych.2019.04.034

**Published:** 2019-08-15

**Authors:** Oliver Pain, Andrew J. Pocklington, Peter A. Holmans, Nicholas J. Bray, Heath E. O’Brien, Lynsey S. Hall, Antonio F. Pardiñas, Michael C. O’Donovan, Michael J. Owen, Richard Anney

**Affiliations:** a Medical Research Council Centre for Neuropsychiatric Genetics and Genomics, Division of Psychological Medicine and Clinical Neurosciences, School of Medicine, Cardiff University, Cardiff, United Kingdom; b Social, Genetic and Developmental Psychiatry Centre, Institute of Psychiatry, Psychology and Neuroscience, King's College London, London, United Kingdom

**Keywords:** ASD, Autism, Colocalization, Expression, Transcriptome, TWAS

## Abstract

**Background:**

A recent genome-wide association study (GWAS) of autism spectrum disorder (ASD) (*n*_cases_ = 18,381, *n*_controls_ = 27,969) has provided novel opportunities for investigating the etiology of ASD. Here, we integrate the ASD GWAS summary statistics with summary-level gene expression data to infer differential gene expression in ASD, an approach called transcriptome-wide association study (TWAS).

**Methods:**

Using FUSION software, ASD GWAS summary statistics were integrated with predictors of gene expression from 16 human datasets, including adult and fetal brains. A novel adaptation of established statistical methods was then used to test for enrichment within candidate pathways and specific tissues and at different stages of brain development. The proportion of ASD heritability explained by predicted expression of genes in the TWAS was estimated using stratified linkage disequilibrium score regression.

**Results:**

This study identified 14 genes as significantly differentially expressed in ASD, 13 of which were outside of known genome-wide significant loci (±500 kb). *XRN2*, a gene proximal to an ASD GWAS locus, was inferred to be significantly upregulated in ASD, providing insight into the functional consequence of this associated locus. One novel transcriptome-wide significant association from this study is the downregulation of *PDIA6*, which showed minimal evidence of association in the GWAS, and in gene-based analysis using MAGMA. Predicted gene expression in this study accounted for 13.0% of the total ASD single nucleotide polymorphism heritability.

**Conclusions:**

This study has implicated several genes as significantly up/downregulated in ASD, providing novel and useful information for subsequent functional studies. This study also explores the utility of TWAS-based enrichment analysis and compares TWAS results with a functionally agnostic approach.

Autism spectrum disorder (ASD) comprises a group of neurodevelopmental disorders characterized by impaired social and communication skills and stereotyped and repetitive behaviors. ASD has a prevalence of 1% (1), with symptoms typically starting in early childhood. Twin studies estimate the heritability of ASD at between ∼65% and 90% 2, 3, demonstrating that genetic differences play an important role in the development of ASD. Common genetic variation is an important component of ASD liability, with a most recent single nucleotide polymorphism (SNP) heritability estimate of 11.8% on a liability scale (assuming prevalence of 1.2%) (4).

Genome-wide association studies (GWASs) are a powerful approach for understanding the role of common alleles in the genetic etiology of traits and disorders and have provided several insights into the etiology of ASD. The most recent and largest ASD GWAS, including 18,381 ASD cases and 27,969 controls, reported three independent loci achieving genome-wide significance (4). Genes associated with ASD were highlighted through proximity to genome-wide significant loci and via joint statistical analysis of variants within gene regions. However, a variant’s proximity to a gene is only one metric for illuminating its functional consequence, and the nearest gene often does not drive the association (5).

Another approach for highlighting genes that underlie GWAS associations is integration of functional data. For example, Grove *et al.* (4) used chromatin conformation data to infer whether significant ASD loci physically interact with the surrounding genes. Alternatively, prior knowledge of variants effecting gene expression, known as expression quantitative trait loci, can be used to infer gene expression changes associated with a given phenotype based on GWAS SNP effects. This is a powerful approach because the vast majority of loci identified through GWASs of complex traits appear to be mediated by altered gene regulation rather than changes in protein coding sequence (6). Several methods exist for inferring associated differential expression from GWAS summary statistics, including Summary-data–based Mendelian Randomization (SMR) (7) and transcriptome-wide association study (TWAS) [as performed by FUSION (8) and MetaXcan (9)]. A key distinction between SMR and TWAS is that TWAS considers the joint effect of multiple SNPs on a gene’s expression and therefore has greater power than SMR when there are multiple expression quantitative trait loci for a given gene 9, 10. In addition to prioritizing genes at genome-wide significant loci, TWAS is able to implicate genes in regions containing no genome-wide significant variants. For example, a recent TWAS of schizophrenia identified 157 significantly associated genes, 35 of which were considered novel as they were >500 kb from a genome-wide significant locus (11). Furthermore, by indicating how the regulation of the implicated gene is affected by associated genetic variation, such studies can more accurately inform functional follow-up investigations and, potentially, therapeutic strategies.

In this study, we carried out a TWAS of ASD to identify gene expression changes associated with these disorders. Using the ASD TWAS results, and a novel adaptation of established statistical methods, we also tested for enrichment within candidate pathways, specific tissues, and stages of brain development. Finally, we estimated the proportion of variance in ASD that is attributable to these TWAS observations.

## Methods and Materials

### Datasets

We performed a TWAS using the publicly available Psychiatric Genomics Consortium + iPSYCH ASD GWAS summary statistics (4) (https://www.med.unc.edu/pgc/results-and-downloads) and 16 sets of gene expression SNP weights (Table 1). SNP weight sets captured gene expression for fetal brain tissue and brain, blood, and adipose tissue in adults. SNP weights for each gene-tissue pair is referred to as a feature. Fetal brain features were derived using gene expression data collected from brain homogenates from 67 fetuses 12 to 19 weeks postconception, and genetically defined to be of European ancestry, collected through the Human Developmental Biology Resource (12). Fetal brain features were derived using the FUSION pipeline (http://gusevlab.org/projects/fusion). CommonMind Consortium (CMC), Netherlands Twin Registry (NTR), Young Finns Study (YFS), Metabolic Syndrome in Men study (METSIM), and Genotype-Tissue Expression project (GTEx) SNP weights were downloaded directly from the FUSION/TWAS website (http://gusevlab.org/projects/fusion/). Analysis of genotypes and gene expression from these datasets has been previously described: CMC (11), NTR, YFS, METSIM (8), GTEx (13), and fetal brain (12). See Supplement 1 for further details on derivation of SNP weights. Less disease-relevant tissues, such as adipose and blood, that were included as cis-expression quantitative trait loci effects are moderately to highly correlated across all tissues (14), and nonbrain datasets have relatively large sample sizes, providing improved power to detect significantly cis-heritable gene expression.

**Table 1:** Descriptive Statistics for SNP Weight Sets in ASD TWAS

Study	Tissue	Type^a^	Individuals^b^	Features^c^	ASD TWAS Significant
O'Brien (12)	Fetal brain	Gene	67	831	2
O'Brien (12)	Fetal brain	Transcript	67	2865 (2295)	6 (5)
CMC	Dorsolateral prefrontal cortex	Gene	452	5379	1
CMC	Dorsolateral prefrontal cortex	Splicing	452	7735 (3297)	1
NTR	Peripheral blood	Gene	1247	2437	0
YFS	Whole blood	Gene	1264	4657	2
METSIM	Adipose	Gene	563	4637	0
GTEx	Caudate basal ganglia	Gene	100	944	0
GTEx	Cerebellar hemisphere	Gene	89	1512	1
GTEx	Cerebellum	Gene	103	2001	2
GTEx	Cortex	Gene	96	1047	0
GTEx	Frontal cortex BA9	Gene	92	928	1
GTEx	Hippocampus	Gene	81	539	0
GTEx	Hypothalamus	Gene	81	602	2
GTEx	Nucleus accumbens basal ganglia	Gene	93	883	1
GTEx	Putamen basal ganglia	Gene	82	633	0
Total	—	—	—	37,631 (13,243)	19 (14)

Numbers in parentheses for fetal brain transcript level and CMC dorsolateral prefrontal cortex indicate the number of unique genes.

ASD, autism spectrum disorder; BA, Brodmann area; CMC, CommonMind Consortium; GTEx, Genotype-Tissue Expression project; METSIM, Metabolic Syndrome in Men study; NTR, Netherlands Twin Registry; SNP, single nucleotide polymorphism; TWAS, transcriptome-wide association study; YFS, Young Finns Study.

a Type indicates what the features for each dataset represent, i.e., gene-level expression, transcript-level expression, or splicing events.

b Individuals in the reference sample used to derive the feature SNP weights.

c Features included in the TWAS for each SNP weight set.

### Transcriptome-wide Association Study

#### Defining Transcriptome-wide Significance

We estimated transcriptome-wide significance as *p* = 4.25 × 10^−6^ using a permutation procedure to account for the correlation between features within and across SNP weight sets (see Supplement 1). For comparison, false discovery rate (FDR)–corrected *p* values were also calculated.

#### TWAS Analysis Using FUSION

TWAS analysis was performed using the FUSION software with default settings (8).

Colocalization was performed to estimate the posterior probability that GWAS and TWAS associations share a causal SNP (see Supplement 1). Colocalization was performed using the coloc R package, version 3.4.1 (R Foundation for Statistical Computing, Vienna, Austria) (15), implemented by FUSION.

In regions containing multiple significant associations, joint analysis was performed to identify conditionally independent associations. This was implemented using FUSION, with genes considered in a joint model if the boundaries overlapped ±0.5 Mb.

To pool evidence of association for each gene across SNP weight sets, the multiple degrees-of-freedom omnibus test was performed using FUSION.

### Derivation of Non–TWAS-Informed Gene-Based Statistics

TWAS has some similarities and differences to gene-based MAGMA analysis (https://ctg.cncr.nl/software/magma) (see Supplement 1). MAGMA’s gene-based analyses were performed using the ASD GWAS summary statistics to enable a direct comparison with the TWAS results. MAGMA analysis was restricted to genes in the TWAS and contained at least one SNP available in the ASD GWAS and 1000 Genomes linkage disequilibrium (LD) reference. The SNP-wise mean model was used in MAGMA to estimate gene associations, a model also employed by other software (PLINK, VEGAS, SKAT). SNPs were assigned to a gene if they were within 10 kb of the gene.

### TWAS-Based Enrichment Analysis

#### Analytical Procedure

TWAS-based enrichment analysis was performed using a novel adaption of a previously established method for GWAS-based enrichment analysis implemented in the software MAGMA (16) (see Supplement 1). In brief, enrichment analysis was performed using linear mixed model regression of TWAS *Z* score on gene set membership, accounting for the correlation between genes due to LD. We analyzed TWAS association results from all 16 SNP weight sets simultaneously to improve genome coverage and reduce the multiple testing burden. The R package lme4qtl was used to fit the linear mixed model (17). The software used for this analysis is publicly available (https://github.com/bulik/ldsc).

#### Gene Set Enrichment Analysis

TWAS results were tested for enrichment across 173 candidate gene sets, including 134 gene sets relevant to various aspects of nervous system function and development (herein referred to as central nervous system gene sets), 38 gene sets that have been previously implicated in ASD specifically (herein referred to as ASD-relevant gene sets), and a gene set containing loss-of-function intolerant genes. The central nervous system, loss-of-function 18, 19, and ASD-related gene sets (20) have been previously described. The FDR method was used to correct for multiple testing across all 173 candidate gene sets.

The comparative analysis using non–TWAS-informed gene-level associations was also performed using MAGMA.

#### Gene-Property Association Analysis

Gene-property analysis estimates the relationship between TWAS associations and continuous gene annotations. Using BRAINSPAN data (21), scores indicating preferential expression of each gene at 19 developmental stages have been calculated (11). Using the mixed model approach described above, the correlation between preferential expression scores for each developmental period and nonzero association gene *Z* scores was then calculated. A significance threshold of *p* < .05/19 was used.

For comparison, gene-property analysis was also performed using the non–TWAS-informed gene-level associations in MAGMA.

#### SNP Weight Set Enrichment Analysis

We also tested for an enrichment of association across the SNP weight sets used in this study to evaluate the importance of each tissue or time point in ASD etiology. Secondary analysis was also performed using only SNP weight sets for the basal ganglia to compare each of the three basal ganglia components.

### Estimating the Proportion of Heritability Mediated by Gene Expression

The proportion of ASD heritability accounted for by the TWAS results from each SNP weight set and all SNP weight sets combined were estimated using stratified LD score regression (22) (https://github.com/bulik/ldsc). FUSION was used to calculate LD scores files that were restricted to SNPs within the TWAS SNP weights, and LD scores were weighted to represent the association between each SNP and predicted gene expression. The total heritability of ASD was estimated using standard LD score regression (23). The proportion of SNP-based heritability accounted for by TWAS was calculated as the TWAS-based heritability divided by the SNP-based heritability.

## Results

### ASD TWAS

**Figure 1:**
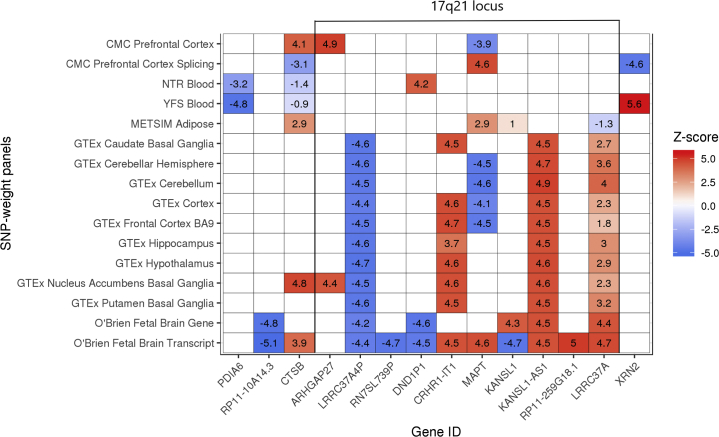
Transcriptome-wide significant genes across single nucleotide polymorphism (SNP) weight sets. Transcriptome-wide significance as a *Z* score is ∼4.6. The direction of effect for splicing and transcript SNP weights should be interpreted with caution owing to the often inverse relationship between alternative transcripts of the gene (11). Blank squares indicate that the gene weights were not available in the target tissue. BA, Brodmann area; CMC, CommonMind Consortium; GTEx, Genotype-Tissue Expression project; METSIM, Metabolic Syndrome in Men study; NTR, Netherlands Twin Registry; YFS, Young Finns Study.

Of the 16 SNP weight sets, 10 revealed transcriptome-wide significant associations, with the fetal brain transcript-level weights returning the greatest number of significant associations (5 unique genes) (Table 1). In total 19 transcriptome-wide significant associations were observed for 14 unique genes (Figure S1 in Supplement 1). Following conditional analysis, 5 independent transcriptome-wide significant associations were observed (Table 2). Many of these associations achieved transcriptome-wide significance across multiple SNP weight sets (Figure 1). Full TWAS association results are in Table S1 in Supplement 2. Colocalization results are available in Table S2 in Supplement 2. When using an FDR-based transcriptome-wide significance threshold, 158 significant features were identified, representing 62 unique genes (Table S1 in Supplement 2).

**Table 2:** List of Independent Transcriptome-wide Significant Loci

Location^a^	MinP (TWAS)^b^	MinP (GWAS)^c^	MinP (MAGMA)^d^	Variance Explained (%)^e^	Jointly Significant^f^	Marginally Significant^g^
chr2:10923518-10952970	1.8 × 10^−6^	1.3 × 10^−4^	4.3 × 10^−4^	94.2	*PDIA6*	*PDIA6*
chr8:8998934-9002945	3.3 × 10^−7^	1.8 × 10^−6^	6.6 × 10^−7^	76.5	*RP11-10A14.3*	*RP11-10A14.3*
chr8:11700033-11725646	2.0 × 10^−6^	3.3 × 10^−6^	1.1 × 10^−6^	96.2	*CTSB*	*CTSB*
chr17:44344403-44346060	5.0 × 10^−7^	4.4 × 10^−6^	1.6 × 10^−7^	99.9	*RP11-259G18.1*	*ARHGAP27*, *CRHR1-IT1*, *DND1P1*, *KANSL1*, *KANSL1*-*AS1*, *LRRC37A*, *LRRC37A4P*, *MAPT*, *RN7SL739P*, *RP11*-*259G18*.1
chr20:21283941-21370463	1.8 × 10^−8^	2.0 × 10^−9^	1.9 × 10^−9^	84.5	*XRN2*	*XRN2*

GWAS, genome-wide association study; SNP, single nucleotide polymorphism; TWAS, transcriptome-wide association study.

a Chromosome and start/stop coordinates of the jointly significant gene.

b Minimum *p* value across SNP weight sets for the jointly significant gene.

c The *p* value for top SNP association ±500 kb of the jointly significant gene.

d The *p* value of most significant gene in MAGMA analysis ±500 kb of the jointly significant gene.

e Proportion of the MinP (GWAS) association explained by the most significant TWAS feature in the region, calculated as 1 – (χ^2^-conditioned GWAS association/χ^2^-unconditioned GWAS association).

f Genes that remain significant after accounting for variance explained by all nearby marginally significant genes.

g Genes that are no longer significant after accounting for variance explained by surrounding jointly significant genes; associations are considered to be dependent if they are with 1 Mb of each other.

#### Chromosome 20 p11.22

**Figure 2:**
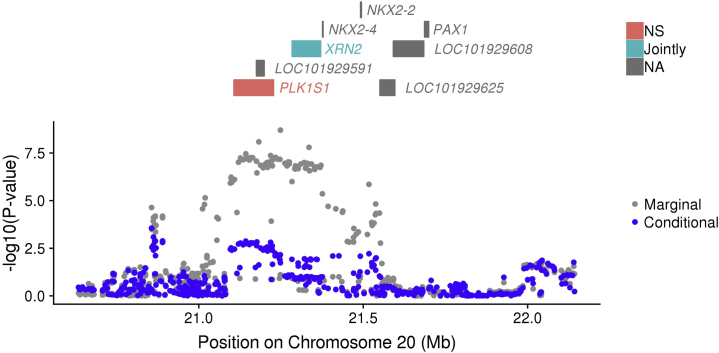
Regional association plot. The top panel shows all of the protein-coding genes or genes in the transcriptome-wide association study. Jointly significant genes are highlighted in blue, nonsignificant (NS) genes are highlighted in red, and genes that were not in the transcriptome-wide association study (NA) are in gray. The bottom panel shows a Manhattan plot of the genome-wide association study data before (gray) and after (blue) conditioning on the jointly significant genes.

The strongest ASD TWAS association was the upregulation of *XRN2* based on YFS blood SNP weights (*p* = 1.80 × 10^−8^). Differential splicing of *XRN2* also showed suggestive significance based on the CMC prefrontal cortex SNP weights (*p* = 4.86 × 10^−6^), and as a result the omnibus test *p* value for *XRN2* was 1.50 × 10^−8^. Colocalization supported model 4 with a posterior probability of 0.966, providing evidence that ASD liability and *XRN2* expression associations are driven by the same causal variant. *XRN2* is within a locus previously associated with ASD at genome-wide significance in the ASD GWAS, with predicted expression of *XRN2* explaining 84.5% of the top SNP association (Table 2; Figure 2).

#### Chromosome 17 q21.31

A cluster of 14 transcriptome-wide significant associations (10 unique genes) were observed within a 1-Mb region on chromosome 17 corresponding to an inversion polymorphism that is common in European populations (24). No single SNP within this region achieved genome-wide significance in the ASD GWAS. The most significant TWAS association in this region was the upregulation of an *RP11-259G18.1* transcript in the fetal brain, explaining 99.9% of the ASD SNP association in this region (Figure S2 in Supplement 1). Features in this region were highly correlated (Figure S3 in Supplement 1) and therefore represent a single association. Although *RP11-259G18.1* showed the strongest TWAS association, colocalization supported Model 4 (same causal variant as ASD) for all transcriptome-wide significant associations in this region.

#### Chromosome 8 p23.1

Two genes, *CTSB* (nucleus accumbens basal ganglia) and *RP11-10A14.3* (fetal brain gene- and transcript-level), located 2.7 Mb apart on chromosome 8, achieved transcriptome-wide significance. When considered in a joint model, both the transcript-level feature for *RP11-10A14.3* and gene-level feature for *CTSB* remained nominally significant, indicating that the signal driving the ASD associations with *RP11-10A14.3* and *CTSB* are broadly independent. Although there are no genome-wide significant SNPs within 500 kb of these genes [previous definition of novel (11)], they are either side of a genome-wide significant locus (rs4841432, chr8:10583506; *p* = 4.4 × 10^−8^) (Figure S4 in Supplement 1). In the joint model, the expression of *CTSB* and *RP11-10A14.3* together explains 60% of the association for this genome-wide significant SNP, demonstrating that these TWAS associations are correlated with the previously identified genome-wide significant association and are therefore not entirely novel. Colocalization provides weak evidence that these association are driven by the same causal variant as ASD, as the posterior probability is greater for model 4 (same causal variant) than model 3 (different causal variant), but individually weak SNP effects result in other models being the preferred model (Table S2 in Supplement 2).

#### Chromosome 2 p25.1

The transcriptome-wide significant association between *PDIA6* and ASD on chromosome 2 is in a genomic region showing minimal evidence of association at the SNP level, with the minimum *p* value being 1.3 × 10^−4^ (±2 Mb of *PDIA6*) (Figure S5 in Supplement 1). Colocalization supports model 4, in which ASD and *PDIA6* share a single causal association.

### Gene Set and Property Analysis

Full competitive gene set enrichment results are available in Table S3 in Supplement 2. No gene sets were significant after FDR correction. Of the 135 central nervous system gene sets, 15 achieved nominal significance, with the most significant being synaptic vesicle, presynapse, and abnormal axon guidance (*p* < .015). Of the 38 ASD-related sets, one achieved nominal significance (Parikshak2013_M16). The M16 coexpression module represents early cortical development, with upregulation of this module starting at 10 weeks postconception (25). The loss-of-function intolerant gene set returned an enrichment *p* value of .194, which supports the notion that mutation-intolerance metrics do not characterize ASD GWAS loci, despite their association with ASD risk genes identified through de novo variant studies (26).

Enrichment results of genes preferentially expressed during one of 19 developmental periods in brain returned no significant associations (Figure S6 in Supplement 1). However, preferential expression during 7 of the first 8 developmental stages (12 weeks postconception to 4 months) were positively correlated with ASD TWAS association, and 7 of 11 later stages (10 months to 40 years) were negatively correlated with ASD TWAS association. This trend suggests that ASD TWAS associations are stronger among genes showing high expression during fetal development.

**Figure 3:**
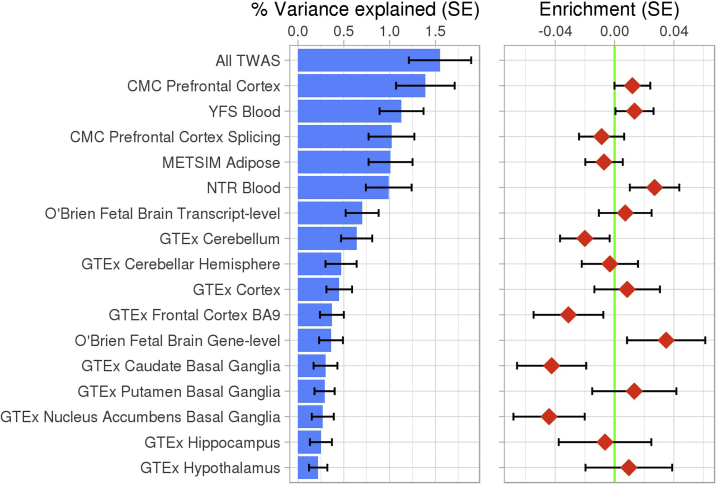
The left panel shows the autism spectrum disorder (ASD) single nucleotide polymorphism (SNP) heritability explained by predicted gene expression on a liability scale. The right panel shows results of competitive gene set enrichment analysis for SNP weight sets (i.e., whether features within each SNP weight set are on average more associated with ASD than compared with features in all other SNP weight sets). BA, Brodmann area; CMC, CommonMind Consortium; GTEx, Genotype-Tissue Expression project; METSIM, Metabolic Syndrome in Men study; NTR, Netherlands Twin Registry; TWAS, transcriptome-wide association study; YFS, Young Finns Study.

Enrichment analysis comparing the mean association of features within each SNP weight set showed no significant enrichment (Figure 3), with fetal gene expression showing the highest level of enrichment (*p* = .09). Secondary competitive analysis of the 3 basal ganglia regions alone showed that gene expression in the putamen region was enriched at nominal significance in comparison with gene expression in the caudate and nucleus accumbens (*p =* .03).

### Comparison of TWAS Results With MAGMA

MAGMA gene association analysis returned similar results to those reported previously (4). Regions containing transcriptome-wide significant associations on chromosomes 8, 17, and 20 also contained significant MAGMA-based associations (±500 kb of significant TWAS feature), although often implicated different genes within the same locus (Tables S4 and S5 in Supplement 2; Figures S7–S12 in Supplement 1). The only transcriptome-wide significant locus in which MAGMA identified no significant associations was that surrounding *PDIA6* on chromosome 2. MAGMA identified significant associations in 2 loci that contained no significant associations in the TWAS. The significant associations in these 2 loci were for *NTM* (11q25) and *MACROD2* (20p12.1) (Figures S9 and S11 in Supplement 1).

Similar to TWAS-based gene set enrichment analysis, MAGMA-based gene set analysis using ASD GWAS summary statistics of candidate gene sets returned no significant associations after FDR correction (Table S3 in Supplement 2). The rank-based correlation between MAGMA- and TWAS-based gene set association results was 0.23.

Gene property analysis for enrichment of genes preferentially expressed during a given period of brain development showed a similar pattern of results as the TWAS-based gene property analysis, with a rank-based correlation of 0.39. Although no developmental stage achieved significance in the MAGMA analysis after Bonferroni correction, preferential expression in 3 fetal stages of brain development were positively associated at nominal significance, and preferential expression in 1 adult brain stage (>19 years) was negatively associated at nominal significance (Figure S6 in Supplement 1).

### Proportion of Heritability

LD score regression estimated the total ASD SNP heritability at .120 (SE = .010; *p* = 4.72 × 10^−32^) on a liability scale assuming a population prevalence of 1.2%. When considering TWAS results from all SNP weight sets together, the heritability was .0155 on a liability scale, and the proportion of ASD SNP heritability explained was 13%. The TWAS-based heritability estimates were all significantly nonzero (Figure 3; Table S6 in Supplement 2). TWAS-based heritability estimates should not be interpreted as a measure of enrichment (see Supplement 1).

## Discussion

This is study has inferred differential gene expression/splicing associated with ASD across a range of tissues using the TWAS method and has provided several novel insights into the etiology of ASD.

This study demonstrates that the previously reported genome-wide significant locus spanning multiple genes within the locus at 20 to 21 Mb of chromosome 20 is linked to significant differential expression and splicing of the gene *XRN2*. Functionally agnostic gene-based analysis in MAGMA also identifies *XRN2* as significant, as reported in this study and previously by Grove *et al.* (4). Moreover, a recent study reported evidence that ASD associated SNPs in this region colocalize with several DNA methylation sites (27), although the consequence of this methylation on surrounding gene expression is unknown. Our data point to differential expression of *XRN2* in the blood and differential splicing in the prefrontal cortex. *XRN2* is an essential nuclear 5′→3′ exoRNase with a multitude of functions in the processing and regulation of RNA molecules. *XRN2* has been identified as an essential gene for the survival of multiple human cell lines 28, 29, 30, and individuals diagnosed with ASD have been shown to have an increased number of deleterious mutations among essential genes (31), again supporting a role for *XRN2* in ASD etiology.

This study also highlighted 13 transcriptome-wide significant genes outside loci achieving genome-wide significance in the corresponding ASD GWAS (±500 kb), 10 of which surround the 17q21 inversion. Five of the 10 significant genes surrounding the 17q21 inversion are also identified as significantly associated using the functionally agnostic region-based approach employed by MAGMA, of which several were previously reported by Grove *et al.* (4). The inversion at 17q21, which has a population frequency of around 20% in Europeans, has been previously highlighted as an ASD susceptibility locus through linkage analysis (32) and family-based GWAS (33). The 900-kb inverted region, which contains many known genes, is marked by extensive LD, complicating identification of the causal susceptibility genes. One study used a fine-mapping approach and implicated *CACNA1G* as a ASD susceptibility gene in the region (34). Results from our TWAS show no evidence of association between ASD and *CACNA1G* expression (*p =* .33 based on CMC prefrontal cortex). Expression of 14 other genes mapping to this region have recently been implicated in the personality trait of neuroticism (12) [genes listed in Tables S8 and S9 of O’Brien *et al.* (12)].

The only locus containing a transcriptome-wide significant gene that is not significantly implicated by either GWAS or MAGMA was *PDIA6* on chromosome 2. This discovery highlights the advantage of TWAS, which incorporates additional functional information of genetic variants as opposed to relying purely on the proximity of SNPs to a gene.

Previous studies using the TWAS approach commonly report associations as novel if the associated feature is outside of genome-wide significant loci in the corresponding GWAS 11, 35. However, given that TWAS is a gene-based approach, it gains power not only from incorporating functional annotations, but also by pooling evidence across multiple genetic variants. Therefore, we have compared the TWAS results to those from the functionally agnostic gene-based approach employed in MAGMA (and other software) to more clearly distinguish the novel insights that TWAS can provide. This comparison demonstrated that 4 of the 5 regions containing independent ASD TWAS associations also contained significant associations identified by the functionally agnostic approach, suggesting that pooling information across genetic variants often highlights regions of novel association. However, the genes implicated often differed between the two gene-based approaches. A key advantage of TWAS is that it considers the functional annotations of associated genetic variants and can therefore provide mechanistic insight into how a regional association is mediated. This is valuable information for subsequent experimental studies aiming to understand the mechanism underlying the genetic association and could be used to improve subsequent gene-level statistical analyses. However, TWAS only assesses genes showing statistically significant cis-heritable expression and is therefore dependent on the sample size of the gene expression reference. As functionally agnostic region-based approaches do not suffer from this limitation, we consider the two approaches to be complimentary, with TWAS as a useful downstream approach for refining and assigning directionality to gene associations.

The two transcriptome-wide significant associations on chromosome 8, *CTSB* and *RP11-10A14.3*, were proximal to a genome-wide significant locus in the corresponding GWAS. MAGMA analysis identified 6 genes within this region achieving significance (Tables S4 and S5 in Supplement 2); however, no gene was identified as significant by both TWAS and MAGMA. Additional support for this locus comes from repeated studies showing duplications in this region (8p23.1-3) in individuals with ASD 36, 37, 38. However, the gene/genes driving this association have not been identified. This TWAS found evidence that *CTSB* is upregulated in ASD across multiple brain tissues. *CTSB* encodes cathepsin B, a cysteine protease reported to mediate exercise enhanced hippocampal neurogenesis and spatial memory (39), and inhibitors of cathepsin B have therapeutic potential for traumatic brain injury (40). Furthermore, treating rodent neuroprogenitor cells with exogenous cathepsin B is associated with differential expression of multiple neurogenesis-related genes (39). These previous findings suggest that differential expression of *CTSB* leads to differences in neurogenesis and neuronal cell death, and therefore is a plausible candidate for ASD. Cathepsin B is also an amyloid precursor protein secretase, and inhibition of it has been reported as a potential therapeutic for Alzheimer’s disease (41). This is interesting, given prior evidence of shared etiology between ASD and Alzheimer’s disease 42, 43. *RP11-10A14.3* is an antisense RNA with an unknown function. Several other genes in this region show suggestive evidence of differential expression/splicing in ASD (Figure S8 in Supplement 1), including *MSRA*, which has been previously associated with schizophrenia (44), *MFHAS1*, and *PINX1*.

Finally, of the TWAS implicated genes, downregulation of *PDIA6* in the blood was significantly associated with ASD. *PDIA6* encodes a member of the protein disulfide isomerase family, which play an important role in protein folding. Owing to their role in protein folding, they have been implicated in several neurodegenerative diseases (45); however, there is little evidence of a connection between protein disulfide isomerases and neurodevelopmental phenotypes. Further research into the potential role of protein disulfide isomerase proteins in ASD is needed. *PDIA6* expression could be assessed only in blood owing to limited power in other tissues and must be replicated in disease-relevant tissues as and when they become available.

Predicted gene expression based on all SNP weights sets separately and together explained a significant amount of variance in ASD liability, collectively accounting for 13% of the ASD SNP heritability. This indicates that gene expression is important in the etiology of ASD.

One other study has applied the TWAS approach to the ASD GWAS summary statistics used in this study (46). Gandal *et al.* (46) infer differential expression in ASD using data from postnatal frontal and temporal cerebral cortex tissue within the PsychENCODE consortium (*N* = 1695), which includes the CMC data used in our study. Gandal *et al.* (46) report 3 loci containing significant associations, all of which overlap with loci highlighted in this study (8p23, 17q21, 20p11). Our results based on nonoverlapping expression data support several of the associations found by Gandal *et al.* (46) and vice versa (Tables S7 and S8 in Supplement 2). One association Gandal *et al.* (46) prioritized is the upregulation of *LRRC37A*, which our study found to be significantly upregulated in fetal brain tissue. A role for *LRRC37A* in ASD during fetal development is further supported by Hi-C (chromatin) interaction analysis from independent fetal brain data (4).

Gandal *et al.* (46) also performed the largest analysis of differential expression in ASD based on postmortem brain tissue. Several of the significant ASD TWAS associations identified in our study also show evidence of differential expression in ASD based on observed gene- and transcript-specific expression from postmortem brain tissue (Tables S9 and S10 in Supplement 2). Significant TWAS associations that are nominally significant based on observed postmortem data include *PDIA6*, *CTSB*, *LRRC37A4P*, *CRHR1-IT1*, *MAPT*, *KANSL1*, and *LRRC37A.*

There are three key limitations to this study. First, TWAS identifies genetic variation that is associated with two outcomes (here, ASD and gene expression/splicing), with subsequent colocalization analysis to determine whether the association is driven by linkage (2 causal SNPs in LD with each other) or pleiotropy (same causal SNP). However, neither TWAS nor colocalization can determine whether the association is causal (the expression mediates the association between SNP and phenotype). Additional studies are required to validate the causal relationship between gene expression changes and ASD. Second, the SNP weights used for predicting differential expression/splicing are based on relatively small sample sizes and therefore cannot infer all features that are cis-regulated across all tissues. As a consequence, there may be features that are important for the etiology of ASD that we are unable to capture currently using TWAS. Finally, the sample size of the ASD GWAS is still relatively small, and therefore power to detect associations is also relatively low.

## Supplementary Material

Supplement 2

Key Resources Table
